# A rare case of term viable secondary abdominal pregnancy following rupture of a rudimentary horn: a case report

**DOI:** 10.1186/1752-1947-3-38

**Published:** 2009-01-29

**Authors:** Bhandary Amritha, Thirunavukkarasu Sumangali, Ballal Priya, Shedde Deepak, Rai Sharadha

**Affiliations:** 1 Department of Obstetrics and Gynecology, Kasturba Medical College, Mangalore, India; 2 Department of Pathology, Kasturba Medical College, Mangalore, India

## Abstract

**Introduction:**

Abdominal pregnancy is a rare event, but one that represents a grave risk to the health of the pregnant woman. An abdominal pregnancy is defined as an ectopic pregnancy that implants in the peritoneal cavity. Early abdominal pregnancy is self-limited by hemorrhage from trophoblastic invasion with complete abortion of the gestational sac that leaves a discrete crater. Advanced abdominal pregnancy is a rare event, with high fetal and maternal morbidity and mortality.

**Case presentation:**

This is a case report of a 22-year-old primigravida with an abdominal pregnancy from a ruptured rudimentary horn. She was diagnosed as a case of term pregnancy with placenta previa with a transverse fetal lie and cervical fibroid and was prepared for an elective cesarean section. Intra-operatively, a live term female baby was extracted from the peritoneal cavity and it turned out to be an abdominal pregnancy from a ruptured rudimentary horn of a unicornuate uterus, which is a very rare condition. Mother and baby were in good condition after such a catastrophic event.

**Conclusion:**

This case illustrates a rare obstetric condition which can be a severe catastrophic condition leading to maternal mortality and morbidity. It is imperative for every obstetrician to have in mind the possibility of abdominal pregnancy, although rare, especially in pregnant patients with persistent abdominal pain and painful fetal movements.

## Introduction

An abdominal pregnancy is defined as an ectopic pregnancy that implants in the peritoneal cavity. Early abdominal pregnancy is self-limited by hemorrhage from trophoblastic invasion with complete abortion of the gestational sac that leaves a discrete crater. Advanced abdominal pregnancy is a rare event, with high fetal and maternal morbidity and mortality. It still remains a diagnostic and therapeutic challenge for every obstetrician and usually occurs after tubal abortion or rupture. Very rarely, it occurs following rupture of a rudimentary horn. We report a rare case of a term viable abdominal pregnancy following rupture of a rudimentary horn.

## Case presentation

**Figure 1:**
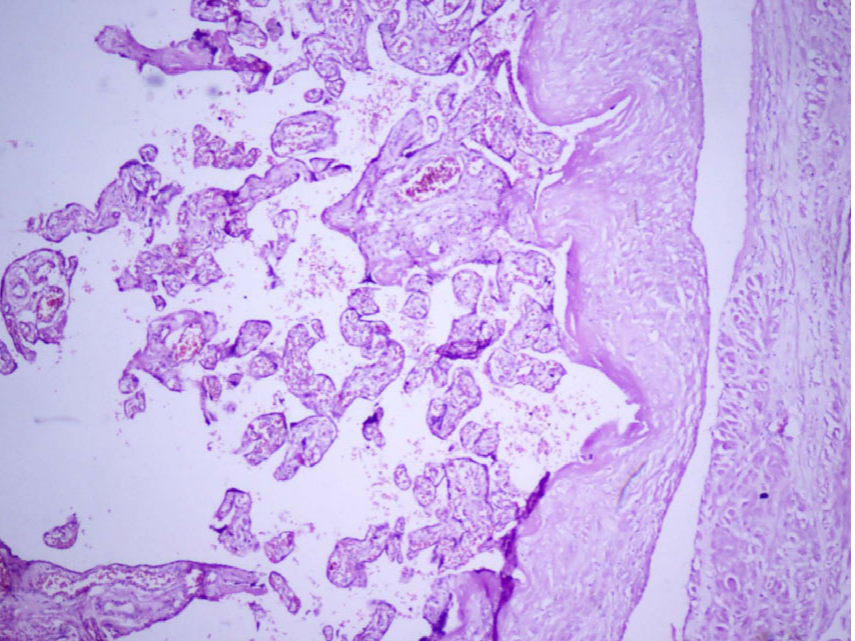
**Histopathological section of placenta showing chorionic villi and bundles of smooth muscle of uterine origin proving the presence of rudimentary horn with placenta**.

A 22-year-old primigravida presented to the obstetrics department at 22 weeks gestation with a painful abdomen of 10 days duration. Her early pregnancy was uneventful and ultrasound examination had not been performed in the first trimester. On examination, her vital signs were stable and tenderness was present in the right iliac fossa and right lumbar region. The height of the uterus corresponded to 28 weeks gestation. Ultrasound showed a fetus of 22 weeks with placenta previa and cervical fibroid. Amniotic fluid was normal. Surgical causes of a painful abdomen were ruled out. The patient was managed conservatively with analgesics and antibiotics and discharged after her pain had subsided. Repeat ultrasound before discharge revealed the same finding. She was lost to follow-up and presented to the outpatient department at 40 weeks of gestation with no complaints for the rest of the antenatal period except for painful fetal movements. It was planned to perform an elective cesarean section for central placenta previa with transverse lie and cervical fibroid. Intra-operatively, as the abdomen was opened, the fetus along with the placenta were found lying in the abdominal cavity and with the left horn of the uterus seen separately lower down in the pelvis. A live term female baby of 3 kg was delivered with good Apgar score. The placenta was attached in part to the ruptured right rudimentary horn deriving its blood supply from it and part of it was attached to the layers of the peritoneum. As the placenta could not be separated from the right rudimentary horn, the placenta along with rudimentary horn and right fallopian tube were removed. The left tube and both ovaries were normal. One pint of blood was transfused. The postoperative period was uneventful and the mother and child were discharged in good condition. The histopathology report showed chorionic villi attached to bundles of smooth muscle of uterine cornu, as shown in Figure 1. Mother and baby were doing well at 6-week follow-up at the outpatient department.

## Discussion

The incidence of abdominal pregnancy is 1 in 10,000 live births, whereas advanced abdominal pregnancy is encountered in 1 in 25,000 births [1]. The maternal mortality rate is 0.5 to 8%, and perinatal mortality ranges between 40% and 95% [2]. A literature review showed that about eight live advanced abdominal pregnancies have been reported so far, but only two cases have been reported which were live and proceeded to term. This case is being reported because of its rarity.

Diagnosis of advanced abdominal pregnancy requires a high index of suspicion. History and physical examination are often inconclusive. Our patient presented only with complaints of painful fetal movements and physical examination showed a transverse fetal lie and closed uneffaced cervix. She had transient unexplained anemia at the time she was in our hospital at 22 weeks for painful abdomen, probably due to rupture of the rudimentary horn. In spite of considerable improvement in technical abilities, absolute diagnosis by ultrasound is missed in half of the cases [1,3]. The following features should alert the sonographer: abnormal relationship among the fetus, placenta, amniotic fluid and uterus, fetal skull or small parts overlying the maternal spine on lateral projection, fetal malpresentation especially transverse lie [4]. In this patient, the normal sized left horn of the uterus mimicked a cervical fibroid and the placenta lying in the peritoneal cavity appeared to be central placenta previa. There was minimal fluid in the right Morrison's pouch which was probably due to rupture of the rudimentary horn and this should be considered an ominous sonographic finding. Magnetic resonance imaging could have been of help in the diagnosis, localizing the area of implantation of the placenta and its vascular supply due to its high resolution [5].

In this patient, the intra-operative findings were indicative of unicornuate uterus with a non-communicating type of rudimentary horn which could have probably ruptured at the time when she presented with painful abdomen, transient anemia and fluid in the right Morrison's pouch. She fortunately continued the pregnancy until term without significant hemorrhage. Maternal deaths associated with abdominal pregnancy result from hemorrhage after inadvertent dislodgement of the placenta. In our patient, part of the placenta was attached to the ruptured rudimentary horn and but most of it lay in the peritoneal cavity attaching itself to the peritoneal layers. It was possible to remove the whole of the placenta along with the rudimentary horn to which it was attached without significant hemorrhage. Removal of the entire placenta has been recommended but if significant hemorrhage occurs, it is safer to leave all or part of the placenta and allow it to reabsorb slowly. If hemorrhage is intractable, ligation of feeding vessels may be attempted. Cases have been reported where hemorrhage was controlled using a medical antigravity suit [3].

In a case report by Desai *et al. *[1], an initial diagnosis of fetal death with placenta previa was made by ultrasound. After repeated failed induction of labor, a careful repeat ultrasound showed a normal sized empty uterus with a macerated fetus in the abdominal cavity.

In three cases reported by Sandberg and Pelligra [3], the diagnosis of abdominal pregnancy was only made intra-operatively as in our case.

In a case report by Harris *et al. *[5], the diagnosis of abdominal pregnancy was suspected by ultrasound but it was confirmed by magnetic resonance imaging (MRI). The area of implantation of the placenta and its relationship to the pelvic organs and the vascular supply could be more closely visualized by MRI.

The delay in diagnosis is mainly due to difficulties in the clinical assessment caused by variance in presentation. A careful examination of the uterine contour in every case may help to avoid misdiagnosis of such a rare and potentially catastrophic presentation.

## Conclusion

The presentation of a pregnant woman with an unusual clinical picture, especially with persistent or recurrent abdominal pain in association with painful fetal movements or intrauterine fetal death, should alert the obstetrician to the possibility of abdominal pregnancy. Expertly performed and interpreted ultrasonography may be the definitive diagnostic technique. It is imperative to consider this diagnosis in the case of such patients and, once discovered, to initiate prompt treatment. Finally, if the entire placental blood supply cannot be ligated, it appears prudent to leave the abdominal placenta *in situ *and to expect spontaneous resorption.

## Consent

Written informed consent was obtained from the patient for publication of this case report and any accompanying images. A copy of the written consent is available for review by the Editor-in-Chief of this journal.

## Competing interests

The authors declare that they have no competing interests.

## Authors' contributions

BA and SD were responsible for the concept, TS wrote the paper, and the manuscript was reviewed and edited by BA and BP. Histopathological confirmation was done by RS. All authors approved the final version.
